# Reading Speed Using the International Reading Speed Texts in a Normal Canadian Cohort

**DOI:** 10.7759/cureus.38196

**Published:** 2023-04-27

**Authors:** Daniel Lamoureux, Sarah Yeo, Vishaal Bhambhwani

**Affiliations:** 1 Ophthalmology, Northern Ontario School of Medicine, Thunder Bay, CAN; 2 Ophthalmology, University of Ottawa, Ottawa, CAN; 3 Surgery, Thunder Bay Regional Health Sciences Centre, Thunder Bay, CAN; 4 Surgery, Northern Ontario School of Medicine, Thunder Bay, CAN

**Keywords:** amblyopia, paragraph reading, international reading speed texts, reading speed, reading

## Abstract

Background

The International Reading Speed Texts (IReST) are commonly used to measure reading speed, which may be affected in many eye conditions. They were originally tested in a younger British population. Our study evaluates IReST in a *normal* Canadian population.

Methodology

A *normal* Canadian cohort in Ontario was prospectively recruited with age >14 years, education >9 years, English as the primary language, and best-corrected visual acuity >20/25 distance and >N8 near in each eye. Participants with eye conditions and neurological/cognitive problems were excluded. Each participant consecutively read two IReST passages (passages 1 and 8). Reading speed in words per minute (WPM) was calculated. One-sample t-test was used to compare our cohort to published IReST standards.

Results

A total of 112 participants were included (35 male, 77 female). The mean age was 40 ± 17 years (14-18 years: 12; 18-35 years: 34; 35-60 years: 53; 60-75 years: 13). The mean reading speed for passage 1 was 211 ± 33 WPM compared to the published IReST standard of 236 ± 29 WPM (p < 0.0001). The mean reading speed for passage 8 was 218 ± 34 WPM compared to the IReST standard of 237 ± 24 WPM (p < 0.0001). Thus, our cohort read slower for both passages compared to IReST standards. The mean reading speed for passages 1 and 8 was the highest for the 14-18-year (231 and 239, respectively) and the lowest for the 60-75-year group (195 and 192, respectively).

Conclusions

Normal older populations have slower reading compared to younger populations. The slower reading in our cohort may also be because the passages were in British rather than in Canadian English. It is important that the IReST is evaluated in different populations to ensure reliable comparison standards for future research.

## Introduction

Reading is an important visual task and affects numerous aspects of daily functioning, including activities related to education, work, household maintenance, and leisure; thus, reading impairments may have negative impacts on an individual’s quality of life [1].

Reading ability varies based on age, education level, and intellectual capacity [2,3] and may also be adversely affected by cognitive and learning disorders such as dyslexia [4]. For eye/visual system pathologies, considerable recent research has focussed on reduced reading speed in individuals with amblyopia and strabismus, macular degeneration, corneal disorders, and glaucoma [1,5-11].

Standardized texts are required to obtain reliable measurements of reading speed in clinical and research settings. Initially, single-sentence reading tests such as the Minnesota Reading Test (MNREAD) [12] and the Radner test [13] were developed for this purpose. However, for measuring reading speed, a whole paragraph of text is preferable to single sentences because reading speed measurements show less variance with longer texts [14]. Furthermore, reading whole paragraphs is closer to the demands of everyday reading. Thus, more recently, the International Reading Speed Texts (IReST) have been developed for longer paragraph reading as a tool for reading, rehabilitation, and low vision research [15-17]. These texts were initially developed in four languages [15] and later expanded to include a wide range of additional languages to allow international multi-language studies, as well as to provide benchmarks for reading speeds in normally sighted readers who read different languages.

It is important that the IReST texts are tested in different populations to ensure that they provide reliable and repeatable measures of reading speed across demographic profiles and can be used for clinical and research-related decision-making. This has been attempted in some populations [18-20]; however, there is a paucity of literature on this subject. This study aims to evaluate reading speed using the English-language IReST charts (developed for British English) in a *normal* Canadian population and compare results with the published IReST standards.

## Materials and methods

A prospective study was conducted to evaluate reading speed using IReST charts for a normal Canadian cohort in Ontario, Canada. Inclusion criteria for the study were age >14 years, education >9 years, English as the primary language, and best-corrected visual acuity >20/25 in each eye for distance and >N8 for near. Exclusion criteria were the presence of eye conditions (other than a refractive error) or neurological/cognitive conditions including learning disabilities which may impact reading speed.

Demographic data collected included participant sex, age, ethnicity, and level of education. The IReST charts in British English were used for reading speed assessment. Each participant sequentially read two passages (passages 1 and 8) of the IReST charts with a break of two minutes between the two passages. These two passages were chosen as they belong to the same IReST performance category, meaning that their mean reading speeds do not differ by more than 10 words per minute (WPM) according to initial studies by the IReST group [17]. Before the start of the reading test, participants were asked to cover the reading passage with their hand, and once instructed to start, to remove their hand and read the paragraph aloud as quickly as possible, as described in the IReST instructions. The reading time was measured using a stopwatch. If a word was skipped, or if an incorrect word was read, this was noted and factored into calculating their reading speed in WPM. Reading speed (WPM) was calculated using the following formula: Reading speed (WPM) = (60/participant time in seconds) × (number of correctly read words in the passage).

The one-sample t-test was used to compare the reading speeds of our cohort to published IReST values. The paired t-test was used to compare the reading speeds of our cohort for passage 1 versus passage 8.

The study was approved by the institutional ethics review board (Thunder Bay Regional Health Sciences Centre Research Ethics Board; approval number: TBRHSC #2020515).

## Results

A total of 112 participants were included (35 males, 77 females). The mean age (±SD) of the cohort was 40 ± 17 years (range = 14-75 years; 14-18 years: 12, 18-35 years: 34, 35-60 years: 53, 60-75 years: 13). The mean years of education (±SD) of the cohort was 15 ± 3 (range = 9-20 years). The ethnicity of the cohort included Caucasian (78, 70%), Indigenous (6, 5%), others (11, 10%), and undisclosed (17, 15%).

The distribution of reading speed in the different age groups of the cohort is detailed in Table 1. As seen in the table, the mean reading speed (±SD) of the cohort for passage 1 was 211 ± 33, and for passage 8 was 218 ± 34. The one-sample t-test was statistically significant for both passage 1 (p < 0.0001) and passage 8 (p < 0.0001), with our cohort reading slower compared to the published IReST values.

**Table 1:** Reading speed data. Reading speeds in different age groups of the study cohort compared to published IReST standards. IReST: International Reading Speed Texts

Serial number	Age (years)	Number of participants	Mean reading speed (±SD) passage 1	P-value for one-sample t-test (compared to IReST standard 236 ± 29)	Mean reading speed (±SD) passage 8	P-value for one-sample t-test (compared to IReST standard 237 ± 24)
1	14–18	12	231 ± 26	0.4830	239 ± 31	0.7917
2	18–35	34	214 ± 30	0.0002	226 ± 32	0.0657
3	35–60	53	209 ± 36	<0.0001	213 ± 34	<0.0001
4	18–60 (total of 2+3)	87	211 ± 34	<0.0001	218 ± 34	<0.0001
5	60–75	13	195 ± 27	0.0001	192 ± 27	<0.0001
6	14–75 (total of 1, 2, 3, and 5)	112	211 ± 33	<0.0001	218 ± 34	<0.0001

With respect to reading speeds in different age groups, the differences in reading speed between our cohort and published IReST values were statistically significant (one-sample t-test) for both passages 1 and 8 for the 35-60 (p < 0.0001 and p < 0.0001, respectively), 60-75 (p = 0.0001 and p < 0.0001, respectively), and 18-60 age groups (p < 0.0001 and p < 0.0001, respectively), and for passage 1 only for the 18-35 age group (p = 0.0002), with our cohort reading slower compared to published IReST values. The reading speeds were not statistically different between our cohort and published IReST values for the 14-18-year age group.

The mean reading speeds for passages 1 and 8 were the highest for the 14-18-year age group (231 and 239, respectively) and the lowest for the 60-75-year age group (195 and 192, respectively).

The difference in reading speed for our cohort between passage 1 and passage 8 (paired t-test) was also statistically significant (p < 0.0001).

## Discussion

Our study results present reading speeds using the IReST charts in British English in a *normal* Canadian cohort (in different age groups) compared to published IReST standards. It is important that *normal* reading speeds are evaluated in different populations across demographic profiles to aid future clinical and research-related decision-making. Our study represents a step in this direction and can be potentially used as a database of *normal* reading speeds with the IReST charts, which are the most commonly used texts for reading speed assessment in clinical practice and research. This is especially important when interpreting reading speeds in individuals with amblyopia and strabismus, macular degeneration, glaucoma, and corneal disorders which are areas of active research interest [5-11].

Our study shows that for our cohort as a whole (aged 14-75 years), reading speeds for both passage 1 and passage 8 were slower compared to published IReST standards. We believe that there are two primary reasons for this difference. First, the original studies by the IReST group were done for subjects aged 18-35 years [15-17]. The published IReST standards reflect *normal* reading speeds in this age group. Slower reading speeds in older individuals in our cohort were partially responsible for reducing the overall mean reading speed of the cohort. Second, the IReST charts are in the British English language. Canadian (and American) English has significant differences compared to British English with respect to vocabulary, phonology, syntax, and semantics [20,21] which may have caused our study participants to read slower compared to published standards. An example of this is the word “greengrocer” in the IReST passage 1, which is not commonly used in Canada. The results of our cohort are a bit different from the study by Morris et al. [20], which showed reading speeds to be comparable to published IReST standards in a Canadian cohort from Quebec. However, this study only recruited university students between 19 and 41 years of age and may have not accounted for differences in reading speed due to the differing age and education levels across a population, as discussed below.

In our study, the only age group which had comparable reading speeds to published IReST standards for both passage 1 and passage 8 was the 14-18-year group. This may be because individuals in this age group routinely do a lot of reading in daily life on account of education and/or work-related activities. In the 18-35-year group, which may still be engaged in reading in routine daily life on account of education/work, slower reading speeds were seen for passage 1 but comparable speeds were seen for passage 8, probably demonstrating a learning effect. This learning effect was also apparent in our study cohort as a whole (aged 14-75 years), with speeds for passage 8 being faster than for passage 1.

In all other age groups besides the 14-35-year subgroups, reading speed for both passages was slower than published standards, with the 60-75-year age group being the slowest. These results are similar to those reported by Morris et al. [22] who found that reading speeds may be slower in older individuals (>60 years), and researchers or clinicians who wish to assess older adults’ reading speed using the IReST charts ought to take this discrepancy into account. Similar results have been seen for the MNREAD charts before [23]. The IReST charts were created using material used for sixth-grade reading for ages 10-12 years and over. Therefore, our study did not include younger participants (children) [15-17].

## Conclusions

It is important that the IReST is evaluated in different populations to ensure reliable comparison standards for future research. Our study demonstrates that populations may differ with respect to *normal* reading speed standards and our results may serve as a database of normal reading speeds in Canadian populations. Age, education levels, and test types may be important factors to consider when interpreting reading speeds. Our study shows that age has a profound influence on reading speed with significantly slower reading speeds in older populations. This effect of age must be taken into account by researchers studying older as well as younger populations. The IReST charts represent a very useful endeavor to standardize reading speed assessments. In our opinion, a fruitful next step could be the development of these charts in Canadian and American English to standardize reading speed tests in North American populations.
